# Endotheliopathy in septic conditions: mechanistic insight into intravascular coagulation

**DOI:** 10.1186/s13054-021-03524-6

**Published:** 2021-03-08

**Authors:** Takashi Ito, Midori Kakuuchi, Ikuro Maruyama

**Affiliations:** Department of Systems Biology in Thromboregulation, Kagoshima University Graduate School of Medical and Dental Sciences, 8-35-1 Sakuragaoka, Kagoshima, 890-8544 Japan

**Keywords:** Sepsis, Disseminated intravascular coagulation (DIC), Endotheliopathy, Extracellular histones, Lipopolysaccharide (LPS), Antithrombin, Thrombomodulin, COVID-19

## Abstract

Endothelial cells play a key role in maintaining intravascular patency through their anticoagulant properties. They provide a favorable environment for plasma anticoagulant proteins, including antithrombin, tissue factor pathway inhibitor, and protein C. Under septic conditions, however, the anticoagulant properties of endothelial cells are compromised. Rather, activated/injured endothelial cells can provide a scaffold for intravascular coagulation. For example, the expression of tissue factor, an important initiator of the coagulation pathway, is induced on the surface of activated endothelial cells. Phosphatidylserine, a high-affinity scaffold for gamma-carboxyglutamate domain containing coagulation factors, including FII, FVII, FIX, and FX, is externalized to the outer leaflet of the plasma membrane of injured endothelial cells. Hemodilution decreases not only coagulation factors but also plasma anticoagulant proteins, resulting in unleashed activation of coagulation on the surface of activated/injured endothelial cells. The aberrant activation of coagulation can be suppressed in part by the supplementation of recombinant antithrombin and recombinant thrombomodulin. This review aims to overview the physiological and pathological functions of endothelial cells along with proof-of-concept in vitro studies. The pathophysiology of COVID-19-associated thrombosis is also discussed.

**Supplementary Information:**

The online version contains supplementary material available at 10.1186/s13054-021-03524-6.

## Background

Blood must be in a fluid state while in blood vessels. However, it must change to a solid state when leaking out of blood vessels. Disseminated intravascular coagulation (DIC) is a pathogenic condition characterized by aberrant activation of coagulation within blood vessels and insufficient activation of coagulation outside blood vessels [1]. This paradoxical condition displays complex clinical manifestations of simultaneous thrombosis and bleeding.

Sepsis is defined as life-threatening organ dysfunction caused by dysregulated host responses to infection [2]. Among dysregulated host responses, including exaggerated inflammation, coagulation, vascular leakage, and tissue hypoperfusion [3], endotheliopathy might play a central role in the pathogenesis of sepsis [4]. For example, disruption of endothelial glycocalyx in septic conditions results in augmented leukocyte adhesion, intravascular coagulation, tissue edema, and dysregulated vasodilatation [5, 6]. In this review, we summarize physiological roles of endothelial cells in maintaining intravascular homeostasis, pathological roles of activated/injured endothelial cells in septic conditions, and proof-of-concept in vitro studies showing these differential roles of endothelial cells.

### Inhibition of coagulation in the intravascular space

**Fig. 1:**
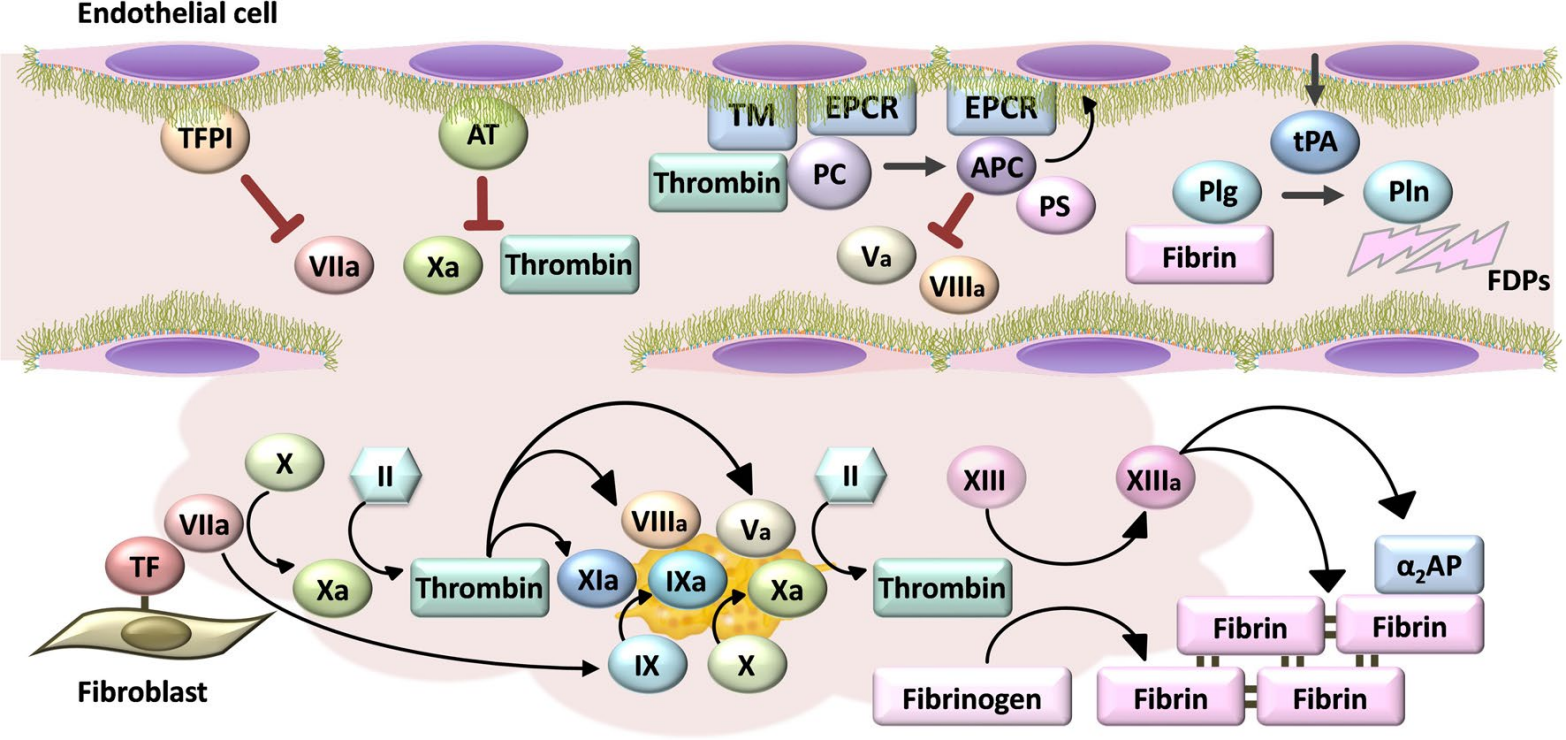
Activation of coagulation in the perivascular space and inhibition of coagulation in the intravascular space. Tissue factor (TF)-bearing fibroblasts, which reside in the perivascular space but not in the intravascular space under physiologic conditions, play an important role in the initiation of hemostasis. An injury to the vessel wall allows plasma coagulation factor VII (FVII) to come into contact with TF-bearing fibroblasts. The FVIIa/TF complex then activates FX and FIX. This results in the generation of a small amount of thrombin, which activates FV, FVIII, FXI, and platelets. This pathway serves as a propagation of coagulation, leading to the generation of large amounts of thrombin and fibrin. Fibrin polymers are then stabilized by FXIIIa, which introduces fibrin–fibrin and fibrin–α2-antiplasmin (α2AP) cross-links. Endothelial cells display heparan sulphate proteoglycans, a component of glycocalyx, which bind and potentiate plasma anticoagulant proteins, including tissue factor pathway inhibitor (TFPI) and antithrombin (AT). Endothelial cells also display thrombomodulin (TM), which promotes thrombin-mediated activation of protein C (PC). Endothelial protein C receptor (EPCR) augments this reaction. Activated protein C (APC) then limits the amplification of coagulation by inactivating FVa and FVIIIa with support from cofactor protein S (PS). Endothelial cells synthesize and release tissue-type plasminogen activator (tPA), which promotes the conversion of plasminogen (Plg) to plasmin (Pln) on the surface of fibrin, leading to the generation of fibrin degradation products (FDPs)

Thrombosis is a frequent complication of blood-contacting medical devices, such as vascular grafts, stents, heart valves, central venous catheters, and extracorporeal circuits [7]. Conversely, this indicates that blood-contacting endothelial cells actively resist thrombosis [8]. Endothelial cells synthesize and display heparan sulphate proteoglycans, a component of glycocalyx, which bind and potentiate plasma anticoagulant proteins, including tissue factor pathway inhibitor (TFPI) and antithrombin [9]. Endothelial cells also express thrombomodulin, which binds thrombin and converts its substrate specificity from procoagulant to anticoagulant. By binding to thrombomodulin, thrombin loses its affinity for fibrinogen, coagulation factor V (FV), FVIII, FXIII, and protease-activated receptors, and instead activates anticoagulant protein C [10]. Endothelial protein C receptor (EPCR) augments this reaction by positioning protein C so that thrombin-thrombomodulin complexes can effectively activate it [11]. Activated protein C (APC) then limits the amplification of coagulation by inactivating FVa and FVIIIa with support from cofactor protein S (Fig. 1). EPCR–APC complexes also elicit cytoprotective effects, including antiapoptotic and barrier stabilizing effects, in endothelial cells. Loss of function of antithrombin, protein C, protein S, and thrombomodulin leads to thrombophilia with varied clinical manifestations, which suggests that these anticoagulant proteins are essential for resistance to intravascular coagulation [12–16]. Furthermore, endothelial cells synthesize and release tissue-type plasminogen activator (tPA) in constitutive and/or regulated manners, potentiating plasmin-mediated fibrinolysis within the vasculature [17].

**Fig. 2:**
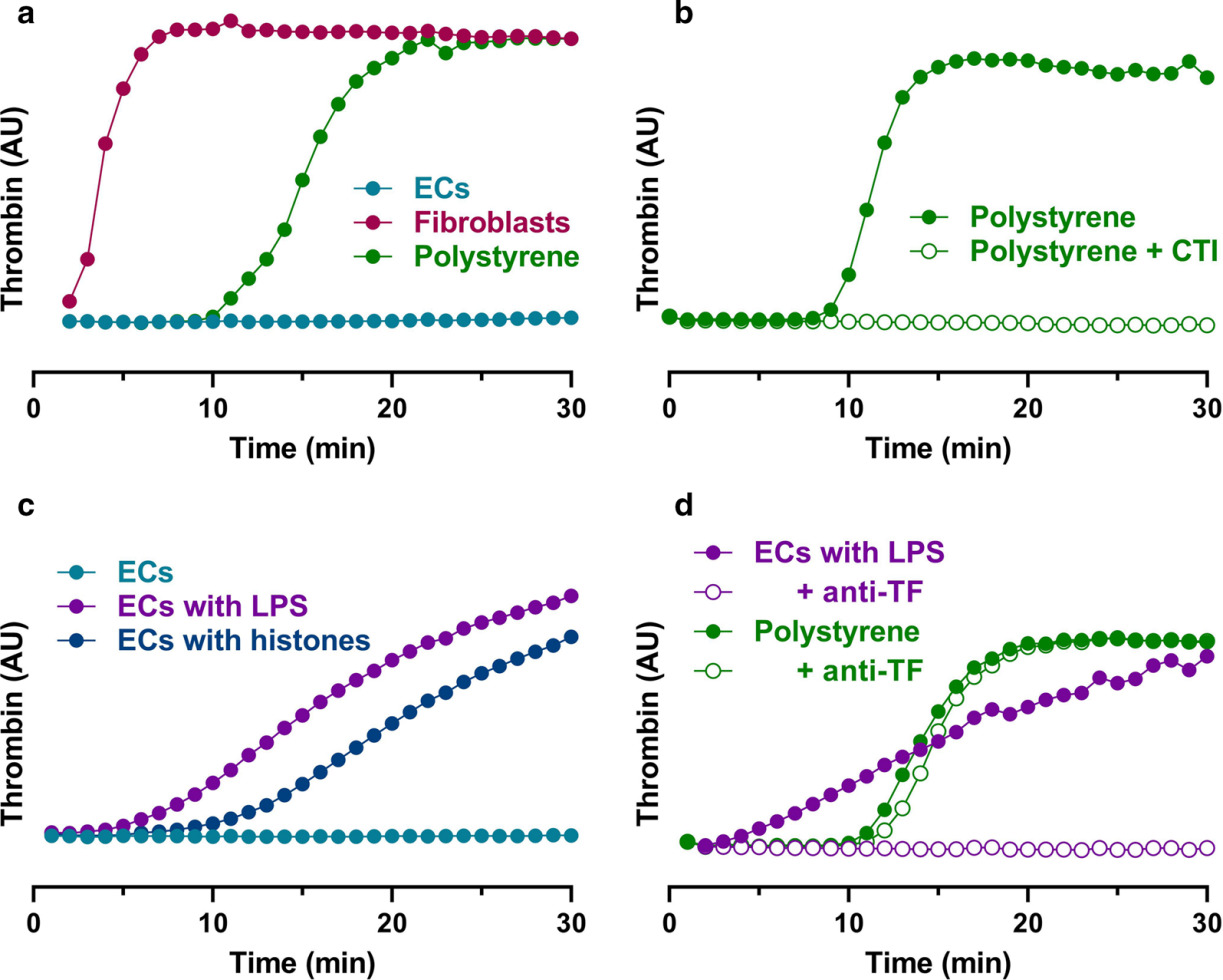
Proof-of-concept in vitro experiments that mimic coagulation on perivascular, intravascular, and artificial surfaces. **a** Thrombin generation on the surface of endothelial cells (ECs), fibroblasts, or polystyrene microplates was monitored for 30 min using a synthetic fluorogenic substrate for thrombin (SN-20) with normal human plasma and CaCl_2_. Thrombin was barely generated on the surface of ECs, rapidly generated on the surface of fibroblasts, and generated after a lag period of about 10 min on the surface of polystyrene microplates. **b** Thrombin generation on the surface of polystyrene microplates was monitored for 30 min either in the presence or absence of corn trypsin inhibitor (CTI), an inhibitor of the intrinsic coagulation pathway. Thrombin generation was completely inhibited by CTI. **c** Thrombin generation on the surface of ECs, either untreated or pretreated with lipopolysaccharide (LPS) or histone H3/H4 for 5 h, was monitored for up to 30 min. Thrombin was generated on the surface of ECs stimulated with LPS or histone H3/H4. **d** Thrombin generation on the surface of fibroblasts or polystyrene microplates was monitored for 30 min, either in the presence or absence of anti-TF antibodies. Thrombin generation on the surface of fibroblasts was completely inhibited by anti-TF antibodies. Detailed methods and results are provided in Additional files 1 and 3

To confirm the concept that an artificial surface is procoagulant while an endothelial surface is anticoagulant, we conducted proof-of-concept in vitro experiments. In these experiments, a synthetic fluorogenic substrate SN-20 was used for monitoring thrombin generation in normal human plasma in the presence of calcium. When plasma was placed on the artificial surface, thrombin was generated after a lag period of about 10 min (Fig. 2a, green). This reaction was completely inhibited by corn trypsin inhibitor, a potent and specific inhibitor of FXIIa (Fig. 2b), which suggests that thrombin generation on the artificial surface is mediated by the intrinsic coagulation pathway. Conversely, thrombin was not generated during the 30-min experimental period when plasma was placed on the surface of cultured endothelial cells (Fig. 2a, blue). These findings indicate that endothelial cells actually confer resistance to the activation of coagulation.

### Activation of coagulation in the perivascular space

Coagulation properties are controlled not only by the coagulation factors but also by the cellular components. Tissue factor (TF)-bearing fibroblasts, which reside in the perivascular space but not in the intravascular space under physiologic conditions, play an important role in the initiation of hemostasis [18]. During the process of hemostasis, an injury to the vessel wall allows plasma coagulation factors, including FVII, to come into contact with TF-bearing fibroblasts (Fig. 1). The FVIIa/TF complex then activates FX and FIX. This results in the generation of a small amount of thrombin, insufficient to generate fibrin but enough to activate FV, FVIII, FXI, and platelets. This pathway serves as a propagation of coagulation, leading to the generation of large amounts of thrombin sufficient for fibrin formation [19]. Fibrin polymers are then stabilized by FXIIIa, which introduces fibrin–fibrin and fibrin–α2-antiplasmin (α2AP) cross-links [20]. In our proof-of-concept in vitro experiments, thrombin was not generated on the surface of endothelial cells (Fig. 2a, blue) but was rapidly generated on the surface of fibroblasts (Fig. 2a, red). These findings indicate that our experimental conditions may mimic rapid coagulation in the perivascular space, with no coagulation within the vasculature and gradual coagulation on the artificial surface.

### Activation of coagulation in the intravascular space in septic conditions

**Fig. 3:**
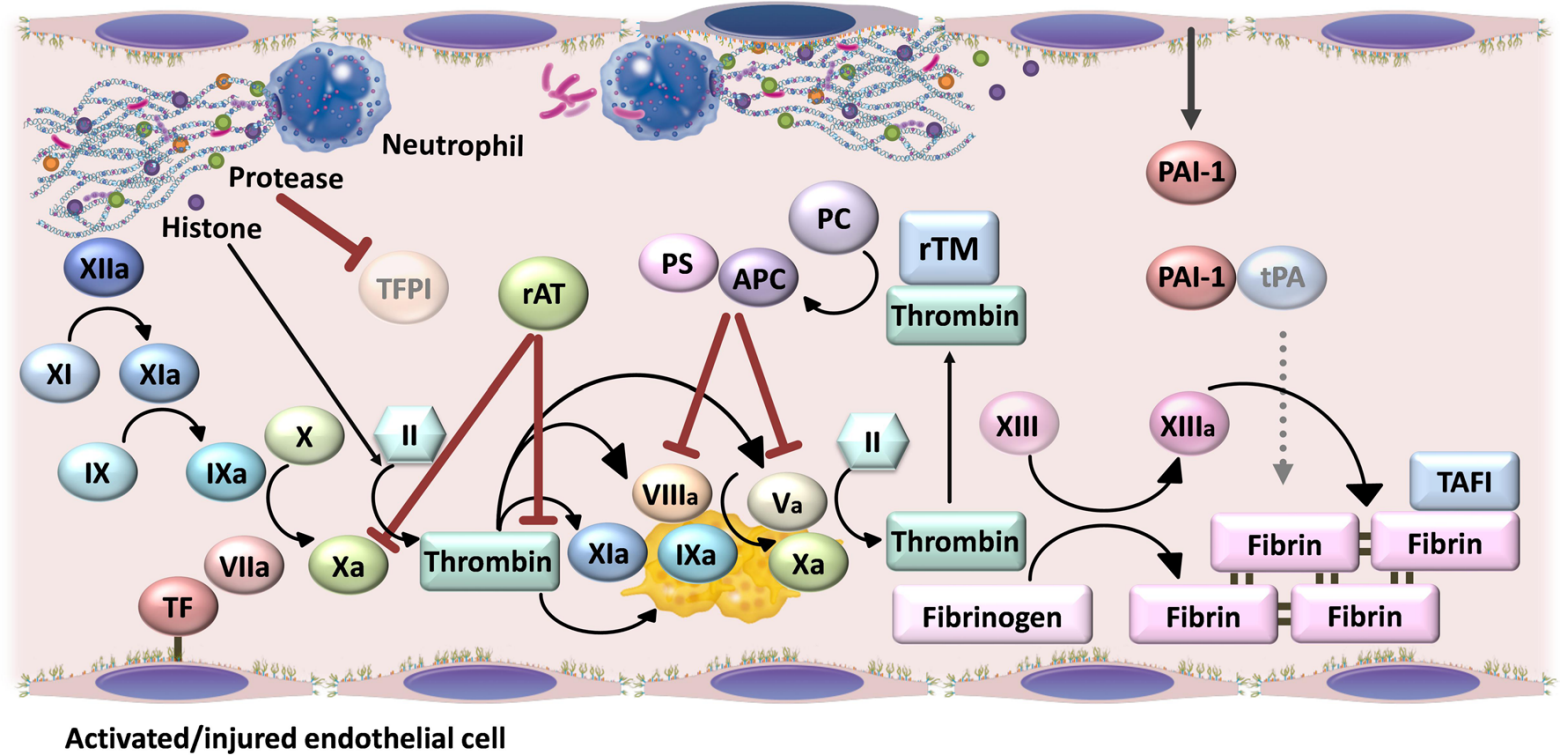
Mechanisms of intravascular coagulation in septic conditions. In septic conditions, anticoagulant potential in the intravascular environment can be compromised because of disruption of endothelial glycocalyx, downregulation of endothelial thrombomodulin, and decline of plasma anticoagulant proteins such as tissue factor pathway inhibitor (TFPI) and antithrombin. In addition, activated leukocytes and endothelial cells provide tissue factor within the blood vessel, leading to intravascular activation of coagulation. Neutrophil extracellular traps (NETs) also provide a scaffold for intravascular coagulation by activating the intrinsic coagulation pathway, facilitating FXa-mediated thrombin generation, and inactivating anticoagulant TFPI. Fibrinolysis inhibitors, such as plasminogen activator inhibitor-1 (PAI-1) and thrombin-activatable fibrinolysis inhibitor (TAFI), are upregulated in septic conditions and exacerbate microvascular thrombosis by preventing fibrin removal. Recombinant thrombomodulin (rTM) and antithrombin gamma (rAT) are potential therapeutic agents that may restore anticoagulant potential within the septic microcirculation. Similar to endogenous thrombomodulin, rTM binds to thrombin to generate activated protein C (APC), which can limit the amplification of coagulation. rAT is an alternative to plasma-derived antithrombin, which traps activated coagulation factors, including thrombin and FXa

While the intravascular space is normally free from thrombosis, intravascular coagulation may occur in septic conditions [21, 22]. In mouse models of infection with *Staphylococcus aureus*, *Escherichia coli*, or lipopolysaccharide (LPS), profound thrombin generation could be observed within the liver microcirculation [22]. Consistent with this, plasma levels of thrombin-antithrombin complex, a clinical biomarker of thrombin generation, were elevated in most patients with sepsis [23]. Activated leukocytes are prominently involved in the pathogenesis of intravascular coagulation as well as overwhelming inflammation in such conditions [24, 25]. In response to microbial stimuli, activated neutrophils release neutrophil extracellular traps (NETs), which can provide a scaffold and stimulus for intravascular coagulation (Fig. 3). NETs comprise DNA, histones, and neutrophil serine proteases, all of which are involved in the activation of coagulation. Negatively charged surfaces of DNA serve as a promoter of the intrinsic coagulation pathway by contact with FXII and FXI [26]. Neutrophil serine proteases inactivate anticoagulant TFPI [27]. Extracellular histones bind to prothrombin, to facilitate FXa-mediated cleavage of prothrombin to release active thrombin [28]. The TF-dependent extrinsic coagulation pathway is also involved in intravascular coagulation, which takes place on the surface of activated endothelial cells and microvesicles originating from activated monocytes [26, 29, 30]. Fibrinolysis shutdown in septic conditions further exacerbates microvascular thrombosis by preventing fibrin removal [31]. Increased levels of plasminogen activator inhibitor-1 (PAI-1) and thrombin-activatable fibrinolysis inhibitor (TAFI) are associated with organ failure and poor outcomes in patients with severe sepsis [32]. Disruption of the endothelial homeostasis by angiopoietin-2 also plays a fundamental role in the pathogenesis of sepsis-associated DIC [4].

To examine whether activated/injured endothelial cells can be procoagulant, we conducted proof-of-concept in vitro experiments. When plasma was placed on the surface of endothelial cells pretreated with LPS or histones, thrombin was generated over time (Fig. 2c, purple and navy). In contrast to the artificial surface, activated/injured endothelial surface exhibited shorter lag time but slower rate of thrombin generation (Fig. 2c, 2d), which suggests that activated/injured endothelial cells might provide both initiators and inhibitors of the coagulation pathway, although the latter might be insufficient compared with normal endothelial cells. Immunoblot analysis revealed that stimulation with LPS resulted in the induction of TF expression and a slight reduction of thrombomodulin expression in endothelial cells (Additional file 1). The induction of TF was responsible for thrombin generation on the surface of LPS-stimulated endothelial cells because treatment with anti-TF antibodies completely diminished thrombin generation (Fig. 2d). Stimulation with histones resulted in the profound reduction of thrombomodulin expression and the exposure of phosphatidylserine to the outer leaflet of the plasma membrane in endothelial cells (Additional file 1). Thus, the effects of LPS and histones on endothelial cells are mechanistically different although both stimulants compromise anticoagulant properties of endothelial cells.

### Impact of hemodilution on procoagulant-anticoagulant balance

**Fig. 4:**
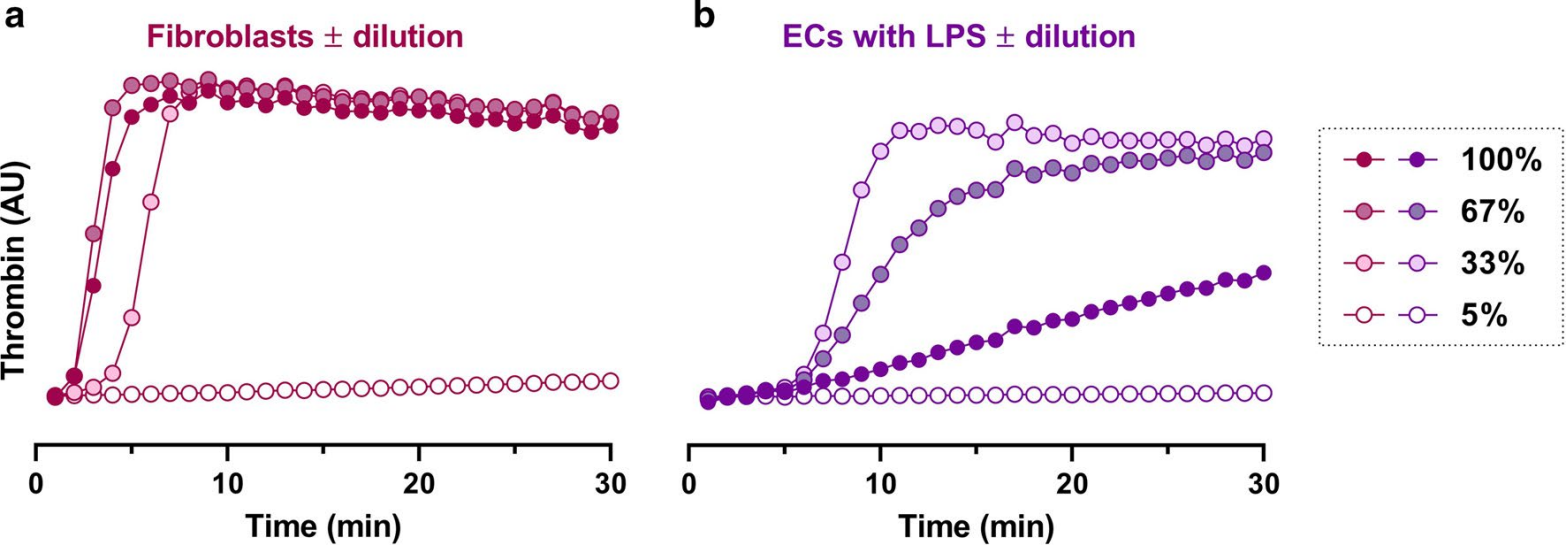
Impact of plasma dilution on procoagulant-anticoagulant balance. Thrombin generation on the surface of fibroblasts (**a**) or endothelial cells (ECs) pretreated with LPS (**b**) was monitored for 30 min using synthetic fluorogenic substrate for thrombin (SN-20) with diluted human plasma and CaCl_2_. For plasma dilution, normal human plasma was diluted with normal saline at a ratio of 2:1 (67% plasma), 1:2 (33% plasma), or 1:19 (5% plasma). While plasma dilution (1:2) delayed the onset of thrombin generation on the surface of fibroblasts, it augmented thrombin generation on the surface of LPS-stimulated ECs. Detailed methods and results are provided in Additional files 2 and 3

Fluid resuscitation decreases plasma levels of coagulation factors, which could eventually result in impaired hemostasis, called dilutional coagulopathy [33]. In our in vitro experiments using fibroblasts, which represent rapid coagulation in the perivascular space, threefold dilution of plasma delayed the time to onset of thrombin generation (Fig. 4a). By contrast, in our in vitro experiments using endothelial cells pretreated with LPS, which represent gradual coagulation in the intravascular space under pathological conditions, threefold dilution of plasma increased thrombin generation (Fig. 4b and Additional file 2). This might have occurred because activation of coagulation was unleashed by the decrease in plasma anticoagulant proteins. The anticoagulant pathways can be far more affected by dilution than the procoagulant pathways in the situation where gradual coagulation takes place [34, 35]. These findings indicate that patients with hemodilution are at risk of intravascular coagulation as well as perivascular bleeding.

### Suppression of coagulation by recombinant thrombomodulin and antithrombin

**Fig. 5:**
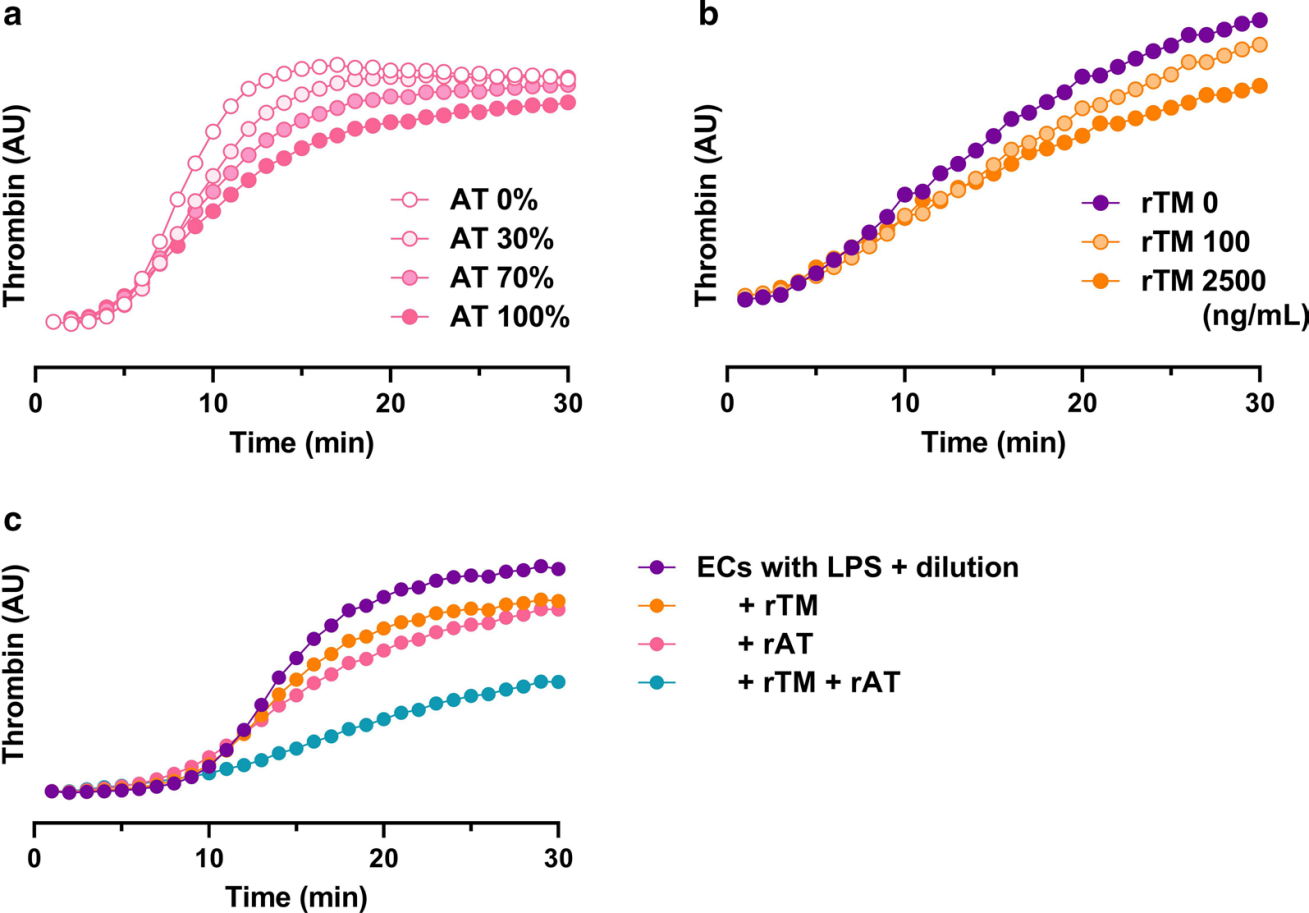
Suppression of coagulation by recombinant thrombomodulin (rTM) and antithrombin (rAT). **a** Thrombin generation on the surface of endothelial cells (ECs) pretreated with LPS was monitored for 30 min using a synthetic fluorogenic substrate for thrombin (SN-20) with antithrombin (AT)-deficient plasma and CaCl_2_. AT-deficient plasma was either unsupplemented or supplemented with rAT to achieve the plasma AT activity of 0, 30, 70, or 100%. Thrombin generation was partially suppressed by the supplementation with rAT. **b** Thrombin generation on the surface of ECs pretreated with LPS was monitored for 30 min using a synthetic fluorogenic substrate for thrombin (SN-20) with normal human plasma and CaCl_2_. Plasma samples were supplemented with 100 or 2500 ng/mL of rTM, which corresponded to minimum and maximum plasma concentrations in the clinical setting. Thrombin generation was partially suppressed by the supplementation with rTM. **c** Thrombin generation on the surface of ECs pretreated with LPS was monitored for 30 min using a synthetic fluorogenic substrate for thrombin (SN-20) with diluted human plasma (67% plasma) and CaCl_2_. Concomitant use of rTM (2500 ng/mL) and rAT (33%) showed additive effects and efficiently suppressed thrombin generation. Detailed methods and results are provided in Additional files 2 and 3

In septic conditions, anticoagulant potential in the intravascular environment can be compromised because of disruption of endothelial glycocalyx, downregulation of endothelial thrombomodulin, and decline of plasma anticoagulant proteins such as TFPI and antithrombin. Recombinant thrombomodulin (rTM) and antithrombin gamma (rAT) are potential therapeutic agents that may restore anticoagulant potential within the septic microcirculation (Fig. 3). Similar to endogenous thrombomodulin, rTM binds to thrombin to generate APC, which can limit the amplification of coagulation without extending coagulation times [36]. rAT is an alternative to plasma-derived antithrombin, which traps activated coagulation factors, including thrombin and FXa [37]. In our proof-of-concept in vitro experiments using LPS-stimulated endothelial cells, thrombin generation was partially suppressed by the supplementation with rAT (Fig. 5a) and rTM (Fig. 5b). Concomitant use of rTM and rAT showed additive effects and efficiently suppressed thrombin generation on the surface of LPS-stimulated endothelial cells (Fig. 5c and Additional file 2). These findings offer valuable insight into the potential of combination therapy with these two drugs because it remains incompletely understood whether rAT may complement anticoagulant effects of the rTM-APC axis or may counteract the rTM-APC axis through inhibition of thrombin-mediated APC generation [38].

### Perspective of anticoagulant therapy in sepsis-associated DIC

A large-scale, randomized, double-blind, placebo-controlled, phase 3 clinical trial, named the KyberSept trial, was undertaken to determine the clinical efficacy of antithrombin in patients with severe sepsis [39]. In this trial, a total of 2314 patients were randomized into two groups to receive either intravenous antithrombin (30,000 IU in total over 4 days) or a placebo (1% human albumin). Although high-dose administration of antithrombin offered no mortality advantage over standard care for sepsis (38.9% vs. 38.7%, *P* = 0.94), there was a trend toward reduced 28-day (37.8% vs. 43.6%, *P* = 0.08) and 90-day mortality (44.9% vs. 52.5%, *P* = 0.03) with antithrombin in the predefined subgroup of patients not receiving concomitant heparin. Furthermore, a post hoc analysis showed that a reduced 28-day mortality with antithrombin was observed in patients with DIC (25.4% vs. 40.0%, *P* = 0.024), whereas no effect was seen in patients without DIC (22.1% vs. 22.2%, *P* > 0.2) [40].

The efficacy and safety of recombinant human APC (rhAPC) in patients with severe sepsis was examined in a large-scale, randomized, double-blind, placebo-controlled, phase 3 clinical trial, named the PROWESS trial [41]. In this trial, a total of 1690 patients were randomized into two groups to receive either intravenous rhAPC (continuous infusion for 96 h) or a placebo. Administration of rhAPC significantly reduced 28-day all-cause mortality (24.7% vs. 30.8%, *P* = 0.005). However, subsequent trials failed to show the efficacy and safety of rhAPC [42, 43], due in part to patient selection and bleeding side effects, leading to its removal from clinical use.

The efficacy and safety of rTM in severe sepsis patients with low platelet counts and prolonged prothrombin times were examined in a randomized, double-blind, placebo-controlled, phase 3 clinical trial, named the SCARLET trial [44]. In this trial, a total of 816 patients were randomized into two groups to receive either intravenous rTM (once daily for 6 days) or a placebo. Administration of rTM did not significantly reduce 28-day all-cause mortality (26.8% vs. 29.4%, *P* = 0.32). A post hoc analysis indicated that the survival benefit with rTM was greater in subgroups with higher levels of thrombin generation at baseline [45]. This might be consistent with the previous finding of high-dose antithrombin, considering that the diagnosis of DIC is based on low platelet counts, prolonged prothrombin times, and increased coagulation biomarker levels [46–48].

A meta-analysis of randomized controlled trials indicated that the survival benefit with anticoagulant therapy was not observed in the overall sepsis population but was observed in the population with sepsis-induced DIC [49]. Thus, the most important issue associated with anticoagulant therapy in septic patients is target selection. Thus far, it is suggested that an optimal target for anticoagulant therapy may be septic patients with DIC and high disease severity. However, this is not based on definitive evidence, and thus, further prospective studies are needed [50].

### Perspective of COVID-19-associated endotheliopathy

Since the identification of severe acute respiratory syndrome coronavirus 2 (SARS-CoV-2) in China in late 2019, the coronavirus disease 2019 (COVID-19) has become pandemic. The clinical manifestation of COVID-19 varies substantially, ranging from almost asymptomatic to life-threatening. Thrombosis is a common and potentially lethal complication of COVID-19 [51, 52]. Endothelial cells are severely injured in alveolar capillaries in fatal COVID-19 cases [53], suggesting that endotheliopathy may play a pivotal role in the pathogenesis of COVID-19-associated thrombosis as in the case of sepsis-associated DIC. However, they differ from each other in several ways. First, thrombocytopenia, prolongation of prothrombin times, and elevation of PAI-1, which are typical in sepsis-associated DIC, are less common in COVID-19-associated thrombosis, at least in the early to mid-stage of this disease [54, 55]. Second, COVID-19-associated thrombosis manifests as not only microvascular thrombosis but also macrovascular thrombosis, such as stroke, myocardial infarction, and venous thromboembolism [56].

Key mechanisms that may cause endotheliopathy secondary to SARS-CoV-2 infection include direct viral toxicity and immune-mediated damage. Given that angiotensin-converting enzyme 2 and transmembrane serine protease 2, which are utilized by SARS-CoV-2 as tools for entry into host cells, are expressed on endothelial cells [57], direct viral toxicity against endothelial cells is a plausible mechanism [53]. Immune-mediated mechanisms may also play a key role particularly in cases where thrombotic organ damage develops in the absence of SARS-CoV-2 viremia or continues to worsen even after most of the virus has been cleared. Dysregulated cytokines, complement, platelets, and neutrophils cooperate to drive a systemic thrombo-inflammatory disorder in COVID-19, as well as traditional sepsis [52, 58].

In addition to these innate immune mediators, autoantibodies may have a significant role in the development of potentially lethal complications in COVID-19 [59, 60]. Compared to uninfected individuals, COVID-19 patients exhibit dramatic increases in autoantibodies against components of their blood vessels, heart, brain, and immune system [61]. Some of the autoantibodies target phospholipids and phospholipid-binding proteins on the surface of endothelial cells, platelets, and neutrophils, tipping the blood–endothelium interface toward thrombosis [59, 62]. Immunoglobulin G isolated from COVID-19 patients has the potential to induce NET release and accelerate thrombosis in mice. Given that the development of autoantibodies generally takes 1–2 weeks, the autoantibody theory might explain some of the delay in the onset of severe complications in COVID-19 [63]. It will be important to understand whether autoantibodies and neutralizing antibodies may persist for a long period after infection, whether these antibodies have direct pathogenic roles, and whether these immune responses have implications for the treatment of COVID-19.

## Conclusions

Endothelial cells confer resistance to the activation of coagulation. Under septic conditions, however, the anticoagulant properties of endothelial cells are compromised, and activated/injured endothelial cells can provide a scaffold for intravascular coagulation. Hemodilution can more profoundly affect anticoagulant pathways than procoagulant pathways and further promote activation of coagulation on the surface of activated/injured endothelial cells. The aberrant activation of coagulation can be suppressed in part by the supplementation with rAT and rTM. These anticoagulants may provide survival benefit in a subpopulation of septic patients who suffer from DIC.

## Supplementary Information

**Additional file 1**. Proof-of-concept in vitro experiments that mimic coagulation on perivascular, intravascular, and artificial surfaces, related to Figure 2.

**Additional file 2**. Impact of plasma dilution, recombinant antithrombin (rAT), and recombinant thrombomodulin (rTM) on thrombin generation, related to Figures 4 and 5.

**Additional file 3**. Supplementary methods for in vitro experiments.

## Data Availability

Not applicable.

## Abbreviations

APC: Activated protein C
DIC: Disseminated intravascular coagulation
LPS: Lipopolysaccharide
NETs: Neutrophil extracellular traps
rAT: Recombinant antithrombin gamma
rTM: Recombinant thrombomodulin
TF: Tissue factor
TFPI: Tissue factor pathway inhibitor
