# Supplementary figures and images for: Endotheliopathy in septic conditions: mechanistic insight into intravascular coagulation
Source: Crit Care. 2021 Mar 8;25:95. doi: 10.1186/s13054-021-03524-6 (PMC7938685; doi:10.1186/s13054-021-03524-6)

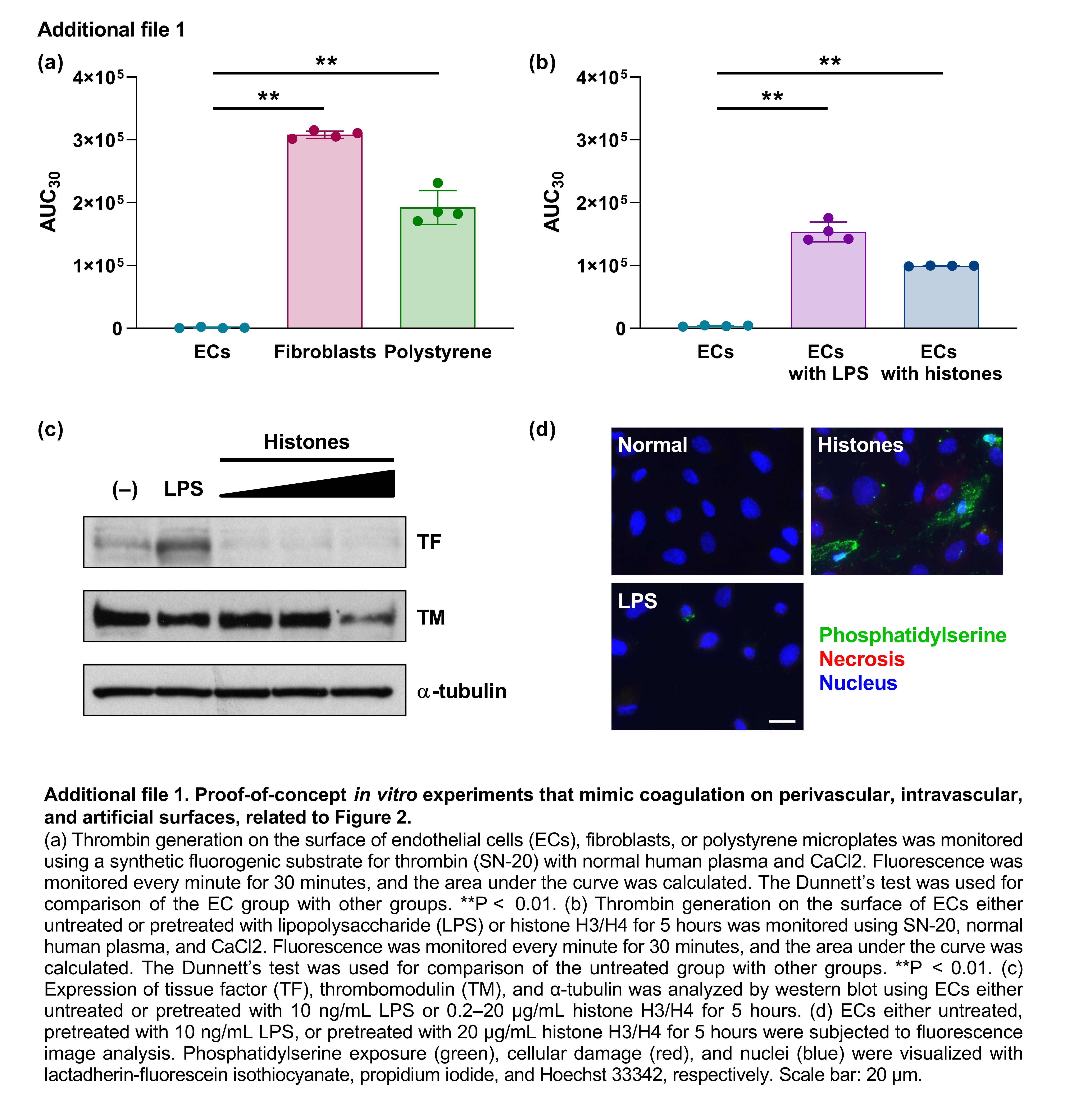

Supplement: Supplementary file 1 — Additional file 1. Proof-of-concept in vitro experiments that mimic coagulation on perivascular, intravascular, and artificial surfaces, related to Figure 2. [file 13054_2021_3524_MOESM1_ESM.jpg]

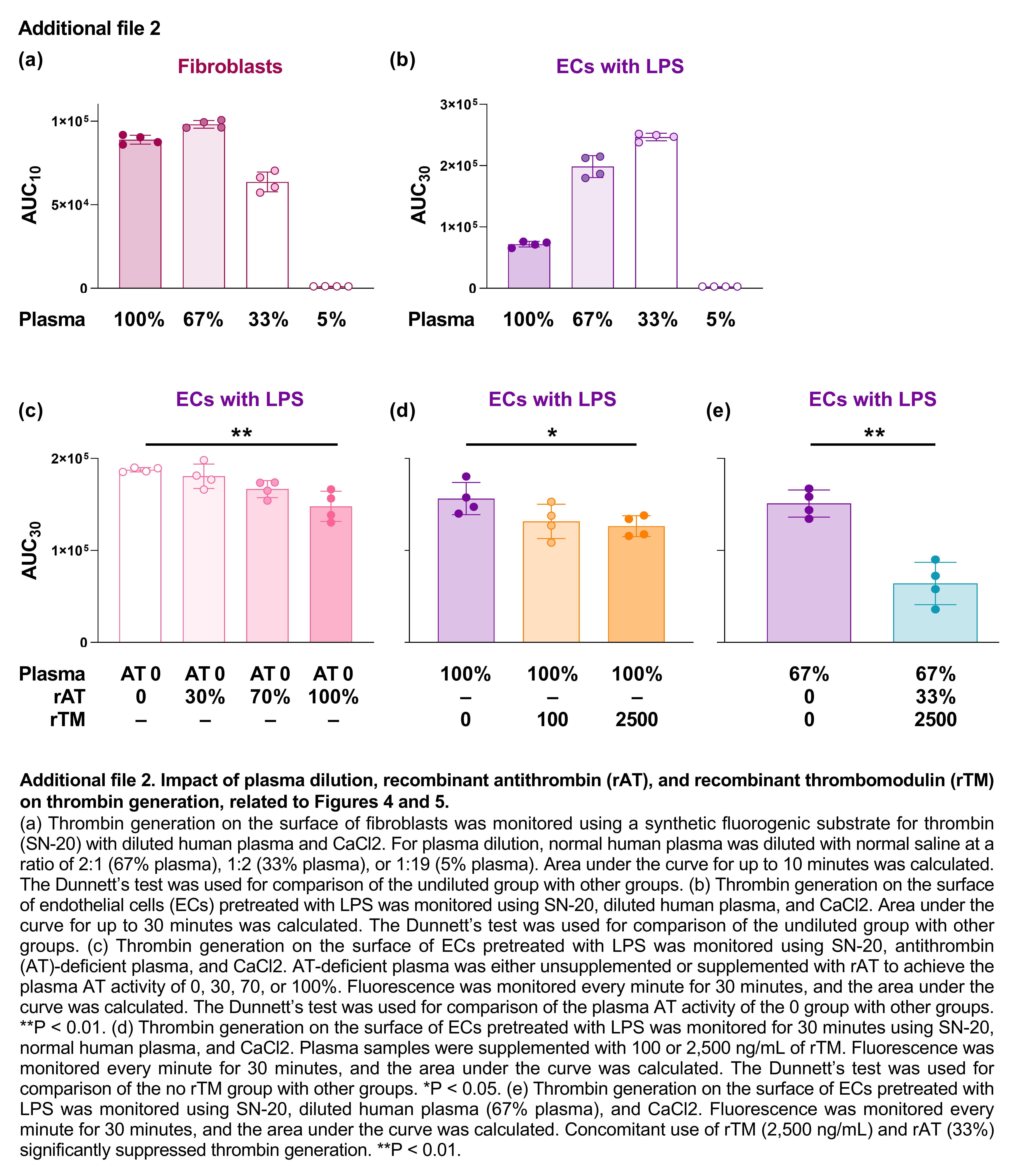

Supplement: Supplementary file 2 — Additional file 2. Impact of plasma dilution, recombinant antithrombin (rAT), and recombinant thrombomodulin (rTM) on thrombin generation, related to Figures 4 and 5. [file 13054_2021_3524_MOESM2_ESM.jpg]
