# Supplementary material for: Endotheliopathy in septic conditions: mechanistic insight into intravascular coagulation
Source: Crit Care. 2021 Mar 8;25:95. doi: 10.1186/s13054-021-03524-6 (PMC7938685; doi:10.1186/s13054-021-03524-6)
Supplement: Supplementary file 3 — Additional file 3. Supplementary methods for in vitro experiments. [file 13054_2021_3524_MOESM3_ESM.pdf]

Supplementary methods for *in vitro* experiments

Cell culture and stimulation

Pooled human umbilical vein endothelial cells (Lonza, Basel, Switzerland) were grown in endothelial growth medium-2 (Lonza). Normal human dermal fibroblasts (PromoCell GmbH, Heidelberg, Germany) were grown in fibroblast growth medium (PromoCell). Cells (1.5×10^5 per well) at passages 2–6 were plated in 96-well polystyrene flat-bottom microplates (AGC techno glass, Shizuoka, Japan) and allowed to grow to confluence prior to performing the thrombin generation assay. In some experiments, cells were pretreated with 10 ng/mL of LPS or 0.2–20 μg/mL histone H3/H4 in Opti-MEM I reduced serum medium (Gibco, Grand Island, NY, USA) for 5 hours.

Thrombin generation assay

A mixture comprising 80 μL pooled normal human plasma (George King, Overland Park, KS, USA), 5 mM fibrin polymerization inhibitor H-Gly-Pro-Arg-Pro-OH (Peptide Institute, Inc., Osaka, Japan), 50 μM synthetic fluorogenic substrate for thrombin (Haematologic Technologies, Inc., Essex Junction, VT, USA), and 4 mM $CaCl_2$ was incubated at 37°C on the surface of polystyrene microplates, normal human dermal fibroblasts, or human umbilical vein endothelial cells. Fluorescence was monitored every minute for 30 minutes, and the area under the curve was calculated for comparison

between groups.

Dilution and anticoagulation

Antithrombin-deficient plasma (Affinity Biologicals, Inc., Ancaster, Canada) or normal human plasma diluted with normal saline at a ratio of 2:1, 1:2, or 1:19 was used in some thrombin generation assays. Citrate concentration was maintained at a constant level of 10.9 mM in the diluted plasma samples. These samples were either untreated or supplemented with rAT to achieve the plasma antithrombin activity of 0, 30, 70, or 100%. In some experiments, plasma samples were supplemented with 100 or 2,500 ng/mL of rTM, which corresponded to minimum and maximum plasma concentrations in the clinical setting. Corn trypsin inhibitor (50 μg/mL, Haematologic Technologies, Inc.) and sheep anti-human TF polyclonal antibodies (100 μg/mL, Haematologic Technologies, Inc.) were used in some thrombin generation assays to specifically inhibit the intrinsic and extrinsic coagulation pathway, respectively.

Western blot analysis

Human umbilical vein endothelial cells either untreated or pretreated with 10 ng/mL LPS or 0.2–20 μg/mL histone H3/H4 for 5 hours were homogenized and used for

western blot analysis. Prepared samples were separated by poly-acrylamide gel electrophoresis, transferred to nitrocellulose membranes, blocked with Block Ace, and incubated with sheep anti-TF antibodies, mouse anti-thrombomodulin antibodies (Santa Cruz Technology, Inc., Dallas, TX, USA), or mouse anti-α-tubulin antibodies (Cell Signaling Biotechnology, Inc., Danvers, MA, USA) at 4°C overnight. The membranes were washed in Tris-buffered saline containing 0.02% Tween-20, and then incubated with horseradish peroxidase-linked secondary antibodies (GE Healthcare, Amersham, UK) followed by detection with an Immobilon Western Chemiluminescent detection system (Merck Millipore, Temecula, CA, USA).

Fluorescence image analysis

Human umbilical vein endothelial cells, either untreated or pretreated with 10 ng/mL LPS or 20 μg/mL histone H3/H4 for 5 hours, were subjected to fluorescence image analysis. Cells were washed in phosphate-buffered saline, labeled with lactadherin-fluorescein isothiocyanate (Haematologic Technologies, Inc.), propidium iodide (MBL Co., Ltd., Nagoya, Japan), and Hoechst 33342 (Dojindo Laboratories, Kumamoto, Japan) at 37°C for 15 minutes, and then observed under a fluorescence microscope BZ-X710 (Keyence, Osaka, Japan).

Statistical analysis

Data are represented as mean (± standard deviation). For comparison of multiple groups, the Dunnett's test was used. $P < 0.05$ was considered statistically significant. Statistical analyses were carried out using GraphPad Prism v8 (GraphPad Software, San Diego, CA, USA).
